# Analysis of Complications and Management After Self-Administration of Medical Termination of Pregnancy Pills

**DOI:** 10.7759/cureus.19730

**Published:** 2021-11-18

**Authors:** Sudhansu Rath, Shilpa Mishra, Ratikanta Tripathy, Sudarshan Dash, Bandita Panda

**Affiliations:** 1 Obstetrics and Gynecology, Kalinga Institute of Medical Sciences, Bhubaneswar, IND; 2 Pharmacology, Kalinga Institute of Medical Sciences, Bhubaneswar, IND; 3 Research and Development, Kalinga Institute of Medical Sciences, Bhubaneswar, IND

**Keywords:** complications, medical termination of pregnancy, suction curettage, ectopic pregnancy, abortion

## Abstract

Background

Medical abortion up to seven weeks of pregnancy by using a combination of mifepristone and misoprostol with careful follow-up is approved by WHO guidelines. But due to the counter sale of medical termination of pregnancy (MTP) pills, in our country, pregnant women have easy access to use them landing upon serious complications. The present study aims to assess the outcome of self-medicated MTP pills in pregnant women.

Method

This prospective observational study includes pregnant women who presented to our hospital for medical assistance due to complications after using the counter of MTP pills without medical consultation. Findings of ultra-sonographic and physical examination were noted along with analysis of subsequent management.

Results

The major complaint at presentation was excessive bleeding (78%). Out of 100 patients, 66% of cases were diagnosed as incomplete abortion, 6% as missed abortion, and 6% as unaffected pregnancy. Ectopic pregnancy was detected in 12% of cases. Sixty patients of incomplete abortion were managed with suction and evacuation and six were supplemented with misoprostol. All patients with ectopic pregnancies were managed surgically.

Conclusion

The majority of the pregnant women who took MTP pills presented with serious complications in the form of bleeding, incomplete/missed abortion, and ectopic pregnancy. Restriction of the over-the-counter dispensation of abortion pills needs to be strictly implemented and knowledge of women regarding the unfavourable outcome of MTP pill intake without proper consultation needs to be improved.

## Introduction

Unsafe abortion is one of the common global reproductive health issues and is very risky, sometimes leading to life-threatening complications. Globally, 19-20 million women face unsafe abortion annually, and in developing countries, it is 18.5 million [1]. In India, the abortion figure is 6.4 million per year, and among them, unsafe abortion is 56%, which accounts for 8-20% of all maternal deaths [1].

As per WHO guidelines, medical abortion is approved for up to seven weeks of pregnancy by using a combination of mifepristone and misoprostol with careful follow-up. It is observed to be completed with a success rate of 90%, avoiding anaesthesia and surgical intervention [2,3]. Various methods have been suggested for evaluation of the completeness of medical abortion such as clinical impression, bimanual pelvic examination, serum β hcg measurement, and endometrial thickness measured by transvaginal sonography (TVS) [4,5]. Complications due to unsafe abortion are incomplete abortion, sepsis, haemorrhage, and uterine perforation along with long-term health issues such as chronic pain, pelvic inflammatory disease, and infertility. It is perceivable that the sale of over-the-counter drugs is unregulated in our country and it includes the medical termination of pregnancy (MTP) pill which is likely to put women at increased risk. The present study was aimed at evaluating the outcome of self-consumption of MTP pills and their management.

## Materials and methods

A hospital-based prospective observational study on complications and management after self-administration of MTP pills was conducted at the tertiary care center in the Department of Obstetrics and Gynaecology, Kalinga Institute of Medical Sciences, and Pradyumna Bal Memorial Hospital, Bhubaneswar for 18 months with due permission from Institutional Ethics Committee and review board. The sample size was 100, which was calculated at a 95% confidence level at a confidence interval by using a survey system [6]. The inclusion criteria were women who presented to seek medical assistance after failure/complication of MTP pill, consumed without medical consultation. A detailed history was collected regarding age, marital status, gestational age, parity, confirmation of pregnancy, duration of pill intake, and presenting complaint. Physical examination and USG findings were noted. Detailed data of further management, e.g., surgical interventions like suction and evacuation, laparoscopy or laparotomy as indicated were recorded. Outcomes and interventions were properly analyzed. Statistical analysis was conducted by using STATA software (StataCorp, College Station, TX).

## Results

The demographic profile shows that majority of patients (42%) presented to the hospital after one to five days of consumption of abortion pills. Some (5%) presented after one month. The period of gestation at the time of pill intake was less than seven weeks in 28% of cases, 7 to 9 weeks in 52% of cases, and between 9 and 12 weeks in 20% of cases as shown in Table 1. Most of the patients (52%) were with gravida 2 followed by 28% who were primigravida and 20% of patients were with gravida more than 2.

**Table 1:** Distribution of patients according to gestational age at the consumption of abortion pills.

Gestational age	Patients (%)
Early pregnancy to 7 weeks	28
7-9 weeks	52
9-12 weeks	20

**Figure 1:**
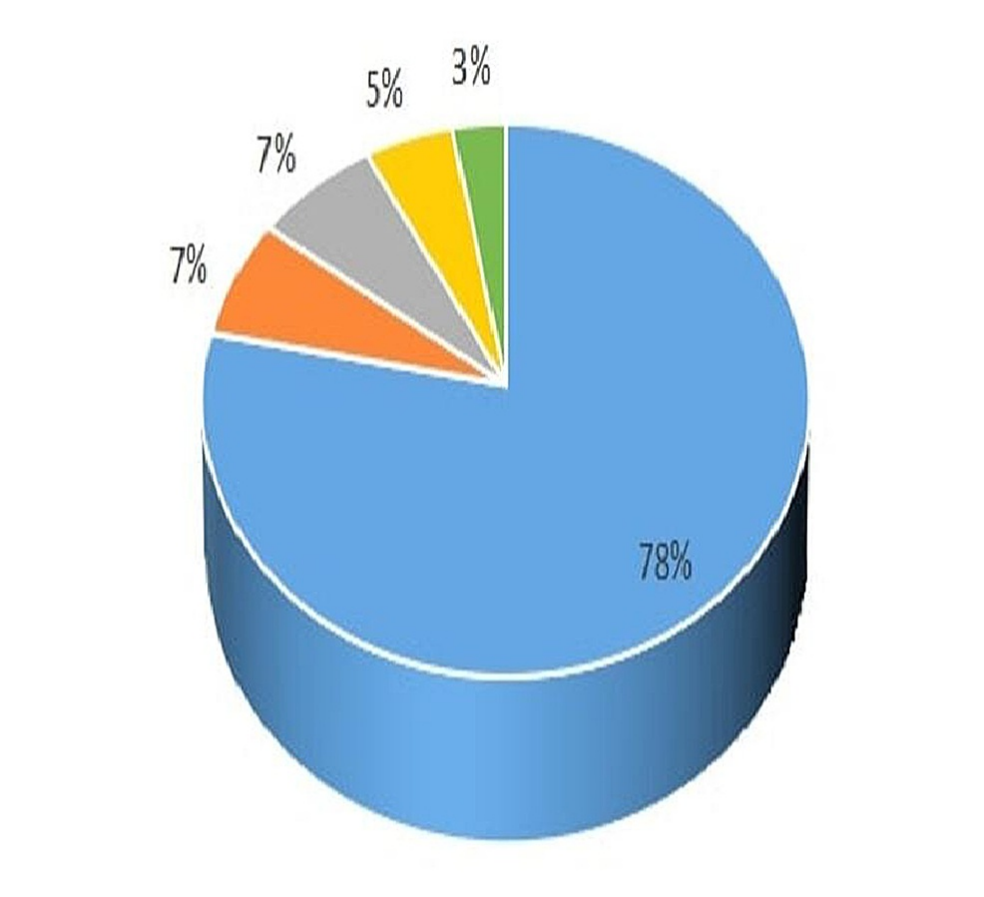
Complaints after consumption of abortion pill. 78%: excessive bleeding per vagina; 7%: abdominal pain; 7%: bleeding with abdominal pain; 5%: non-expulsion of products of conception; 3%: irregular bleeding per vagina.

The main complaint was excessive bleeding per vagina as observed in 78% of patients. Bleeding with abdominal pain and only abdominal pain were encountered in 5% and 3% of patients, respectively. Non-expulsion of products of conception was reported by 7% cases. Symptoms of irregular bleeding per vagina were reported by 7% (Figure 1). A level of haemoglobin less than 10 g/dl was recorded in 50% of cases. Among them, 12% had less than 7 g/dl. Twenty-two patients required blood transfusion. Among these 22 patients, two or more units of transfusion were required in 17 patients as shown in Table 2.

**Table 2:** Requirement of blood transfusion in patients with 21 severe anaemia in 22 cases.

Requirement of blood for blood transfusion	No. of patients
1 unit	5 (22.7%)
2 units	13 (59.1%)
3 units	4 (18.2%)

**Figure 2:**
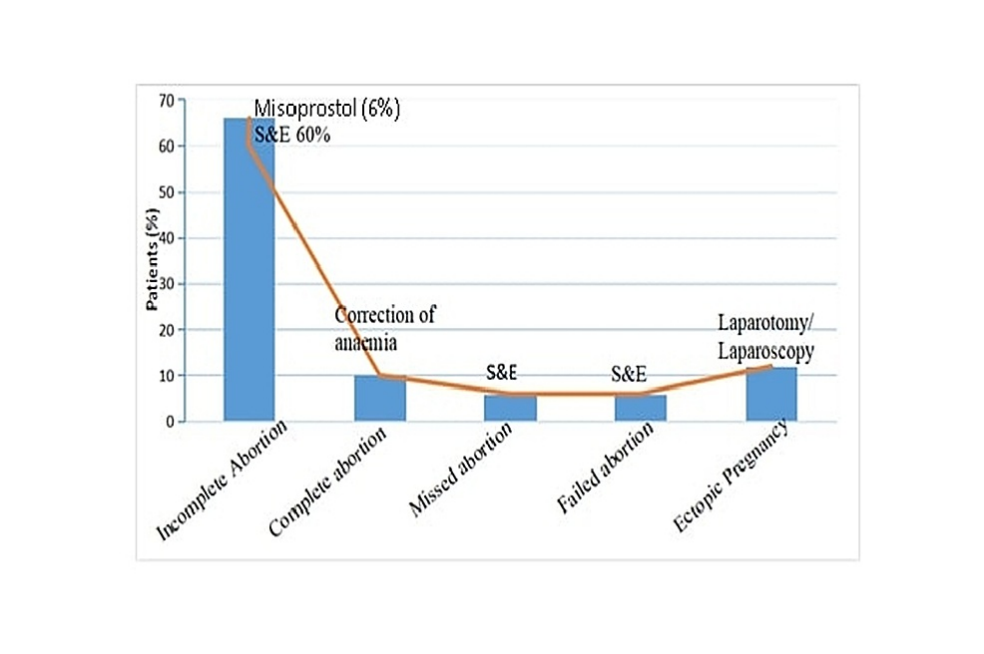
Outcome of abortion pill consumption without medical consultation and management after hospital presentation. Complications after abortion pill consumption management as per complications after hospital presentation.

A total of 66% were diagnosed as having an incomplete abortion, 6% of patients had missed abortion and 6% of patients’ pregnancies were unaffected, whereas 12% of patients were diagnosed as having an ectopic pregnancy. Complete abortion with anaemia was observed in 10% of cases shown in Figure 2.

Management of each complication was handled with procedures as clinically applicable. Most of the patients (60%) with incomplete abortion were treated with suction and evacuation and the rest (6%) were treated with misoprostol shown in Figure 2. All missed abortions and failed abortions were managed with suction and evacuation, whereas all ectopic pregnancy cases were managed with laparotomy/laparoscopy. In the present study, the majority of the patients (74%) had a hospital stay of one to two days followed by three to five days (24%) and five to seven days (2%). All the patients in our study were discharged within a maximum time period of one week in a stable condition with adequate contraceptive counselling.

## Discussion

Self-administered abortion or abortion pill by an unskilled person is considered to be unsafe abortion by WHO. Unsafe abortion mostly leads to complications like sepsis, uterine perforation, cervical trauma, ectopic pregnancy, and incomplete abortion. It is the major reason for maternal morbidity and mortality and has become a global health issue. Medical abortion is a secure alternative to surgical abortion but self-administration without professional consultation is a serious issue [7]. The present study analyzed the problems faced in medical methods of termination of pregnancy after self-administration without medical consultation in 100 patients.

Most of the complaints were excessive bleeding per vagina (78%) followed by bleeding with abdominal pain (5%) and only abdominal pain (3%). Other symptoms like non-expulsion of products of conception and irregular bleeding per vagina were observed in 7% of patients. This result is similar to Nivedita et al. and Giri et al. Excessive bleeding per vagina and abdominal pain are two dominating symptoms [8,9].

In the present study, the gestational age at the consumption of abortion pills in 3228 (28%) patients were early pregnancy (up to seven weeks) while in 52 (52%) and 20 (20%) patients had consumed abortion pills at 7-9 weeks and 9-12 weeks of pregnancy, respectively. Another study found 40% of the women had consumed the pills after nine weeks of gestation [8]. Sarojini et al. study showed the highest number of women (64.4%) took the abortion pills beyond the recommended period of gestation [10]. All these studies are adding to the fact that consumption beyond recommended 63 days limit is quite prevalent in the unsupervised and 35 uncounselled users of the MTP pill, thereby increasing the complication and risk. Another study concluded that efficacy decreases with increasing gestational age (p < 0.001) and for gestations ≤49 days, mean rates of complete abortion were 94-96% [11].

The incidence of anaemia among pregnant women ranges from 33% to 89% and is a direct or indirect cause of 39.20% maternal mortality [12]. Around 50% of total women in our study group had a hemoglobin of less than 10 g/dl with 40.12% having severe anaemia (less than 7 g/dl). More than 50% of anaemic patients required blood transfusion of 2 units of whole blood for the correction of anaemia. The similar result have been reported by another study [9]. Acute blood loss following unsupervised MTP pill intake requiring blood transfusion is a real cause of concern.

Sixty-six percent of women were diagnosed as having an incomplete abortion and 10% as having a complete abortion with anaemia. While the percentage of missed abortions and failed abortions were 6% each. The incidence of incomplete abortion in our study was also comparable to that of 41 other studies [8,9]. There were three on-going pregnancies (3.0%, 95% confidence limits (CL) 0.6-8.6) and four incomplete abortions with MTP (4.0%, 95% CL 1.1-10.0) [12]. In the present study, ectopic pregnancy was detected in 12% of patients due to not having undertaken proper health-care counselling and bimanual or USG examination before intake of MTP Pill. Underdiagnosis of ectopic pregnancy can lead to potentially serious consequences in patients who have taken these pills without prior confirmation of intrauterine gestation [13].

Surgical management by suction and evacuation was needed in 72% of women, including incomplete abortions, missed abortions, and unaffected pregnancies. Six percent of the patients with incomplete abortions were given supplementary medical management with misoprostol. Ten percent of women who had complete abortions needed blood transfusions. Surgical management by suction and evacuation or misoprostol administration along with blood transfusion for correction of anaemia was reported by a study for incomplete abortion [8]. Giri et al. managed some cases of incomplete abortion by repeat administration of mifepristone and misoprostol. [9]. In the study conducted by Sarojini et al., 72.2% of women had an incomplete abortion, 9.6% had missed abortion, and 8.7% had a complete abortion. There were two cases (1.9%) of ruptured ectopic pregnancies [8]. We resorted to laparoscopy in half of the ectopic cases.

The overall frequency of infection after medical abortion was <1% compared to surgical methods when done under prescribed settings [14]. But serious infections like fatal Clostridium infections have been reported following medical abortions [15]. Complications of sepsis tend to be higher in women undergoing unsafe surgical abortions. In the present study, none of the women presented with sepsis. Sharma et al. conducted a study in a rural population which concluded that patients presented with features of sepsis were managed with IV antibiotics along with suction and evacuation [16].

## Conclusions

Unsupervised use of the MTP pill is associated with complications like higher chances of incomplete abortion, failed abortion, and ruptured ectopic pregnancy. Consequent profuse vaginal and intra-abdominal haemorrhage can be life-threatening. It can be avoided only by the enforcement of a strict ban on the over-the-counter dispensation of abortion pills. The creation of public awareness regarding the ill effects of such misuse needs to be a priority as a part of the safe reproductive health programme. The limitation of our study is the inability to assess reasons for self-administration which may be related to privacy and confidentiality outside health care set up. Peer influence and spouse pressure are also factors to consider. It is intended to study these social aspects in our future studies.
